# Band Structure and Quantum Transport of Bent Bilayer Graphene

**DOI:** 10.3390/ma15238664

**Published:** 2022-12-05

**Authors:** Xue Wang, Lei Xu

**Affiliations:** 1 Xinjiang Key Laboratory of Solid State Physics and Devices, Xinjiang University, Urumqi 830046, China; 2 Center for Theoretical Physics, School of Physical Science and Technology, Xinjiang University, Urumqi 830046, China

**Keywords:** bent bilayer graphene, edge and interface state, weak quantum spin hall phase

## Abstract

We investigate the band structures and transport properties of a zigzag-edged bent bilayer graphene nanoribbon under a uniform perpendicular magnetic field. Due to its unique geometry, the edge and interface states can be controlled by an electric field or local potential, and the conductance exhibits interesting quantized behavior. When Zeeman splitting is considered, the edge states are spin-filtered, and a weak quantum spin Hall (WQSH) phase appears. In the presence of an electric field or local potential, a WQSH-QH junction or WQSH-spin-unbalanced QSH junction can be achieved, respectively, while fully spin-polarized currents appear in the interface region. Zeeman splitting lifts the spin degeneracy, leading to a WQSH around zero energy with a quantized two-terminal conductance of 4e^2^/h, which is robust against weak nonmagnetic disorder. These results provide a way to manipulate the band structures and transport properties of the system using an electric field, local potential, and Zeeman splitting.

## 1. Introduction

Graphene, a two-dimensional hexagonal crystalline material, has been successfully fabricated experimentally since 2004 [1,2]. Its unique electronic properties [3] and high electron mobility [4], as well as a series of other physical properties, have rapidly made graphene one of the most popular research objects in the field of condensed matter physics. The linear dispersion relation of graphene near zero energy [5,6] leads to the peculiar quantum Hall (QH) effect [7,8], and this effect can be characterized by chiral edges. Monolayer graphene has a unique “half-integer” QH effect, with a quantum conductivity of $\sigma_{xy}=4\left(n+1/2\right)e^{2}/h$ [7,9,10]. The additional layer symmetry leads to an eightfold degenerate level of the zero Landau level of bilayer graphene [11,12,13]. As a result, bilayer graphene exhibits an integer QH effect [14] with a quantum conductance of $\sigma_{xy}=4ne^{2}/h$. Two kinds of quantum spin Hall (QSH) effects in graphene have been theoretically predicted in different scenarios. One is the time-reversal symmetry protected QSH effect caused by intrinsic spin-orbit coupling [15,16]. The other is the time-reversal symmetry broken QSH effect due to intrinsic spin-orbit coupling combined with exchange fields [17] or magnetic fields [18]. Moreover, the CT-invariant QSH effect in ferromagnetic graphene is also proposed, where C is a charge conjugation operation and T is a time reversal operation [19,20].

In experiments, it is easier to obtain bent graphene than monolayer or bilayer planar graphene, and because of its material properties, it can be bent into three-dimensional structures [21,22] without degrading its structural properties and electron transport after bending [23,24,25]. Therefore, energy band studies on bent graphene have recently attracted great interest from researchers. In previous work, bent monolayer graphene has been studied, and many valuable results have been found, such as the interface state appearing in the folded region of bent monolayer graphene [26,27,28], giving rise to some new transport properties in bent graphene [29,30,31]. The effect of the jump integral after bending curvature correction and the width of the bent region have been investigated in bent monolayer graphene, and the results show that they hardly affect the spatial distribution of the edge and interface states [32]. Although this field of bent monolayer graphene is developing rapidly, the properties of bent bilayer graphene in terms of electric and magnetic field modulation are not yet fully understood. An in-depth study of the transport properties of bent bilayer graphene will contribute to a deeper understanding of the special physical properties of graphene, such as the photoelectric effect and the two-terminal conductance.

**Figure 1:**
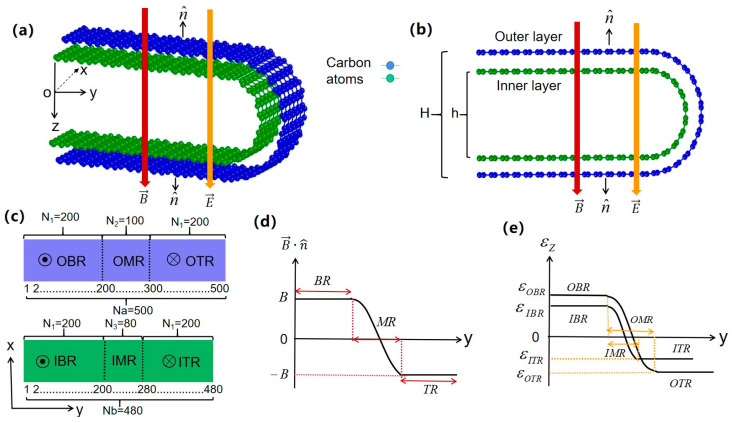
(**a**) Schematic 3D structure of the ZBBGN. The ZBBGN is bent along the y-direction with a uniform electric and magnetic field ($\vec{E}$ and $\vec{B}$) perpendicular to the xy plane. (**b**) Side view of the ZBBGN. H (h) is the interlayer distance of the outer (inner) bent graphene nanoribbon. The ZBBGN is Bernal-stacking. (**c**) Topological equivalence diagrams corresponding to the bent graphene nanoribbon. The OBR (IBR), OMR (IMR), and OTR (ITR) correspond to the bottom plane region, middle bent region, and top plane region of the outer (inner) bent graphene nanoribbon, respectively. Na (Nb) represents the length of outer (inner) layer. N_1_ represents the width of the OBR, OTR, IBR, and ITR. N_2_ and N_3_ represent the widths of the OMR and IMR, respectively. (**d**) The effective magnetic field distribution in different regions of the ZBBGN. (**e**) The effective potential energy distribution in different regions of the sample.

In this work, we consider the zigzag-edged bent bilayer graphene nanoribbon (ZBBGN), as shown in Figure 1. Two flat graphene nanoribbons are bent along the y-direction to form U-shaped nanoribbons with both inner and outer layers bent to 180°. The inner and outer bent graphene nanoribbons are divided into three regions, the top planar region (TR), the bottom planar region (BR), and the middle bent region (MR), with electric and magnetic fields ($\vec{E}$ and $\vec{B}$) in the z-direction and perpendicular to the planar region. The transport properties of ZBBGN are systematically investigated by the tight-binding model and Green’s function method under local potential, and perpendicular electric and magnetic fields. Under a fixed electric field or local potential, the two-terminal conductance and energy bands are no longer symmetric with respect to zero energy. Taking into account the Zeeman effect, the weak QSH (WQSH) and QH phases coexist in the BR and TR, resulting in fully spin-polarized currents in the MR. In the presence of local potential, a WQSH phase can appear in the BR or TR so that a coexisting phase of the WQSH phase and the spin-unbalanced QSH phase appears in both planar regions, while a single spin-polarized current appears near the boundary of the MR. It is also confirmed that the time-reversal symmetry breaking WQSH phase remains robust under weak nonmagnetic disorder.

## 2. Model and Methods

The tight-binding model of bent monolayer graphene in the presence of a uniform perpendicular magnetic field $\vec{B}=\left(0,0,B\right)$ is given by

(1) $$\begin{array}{c} H_{BMG}=-t\underset{\langle i,j\rangle ,\sigma }{\sum }e^{i\emptyset_{ij}}\left(C_{i,\sigma }^{+}C_{j,\sigma }+H.C.\right)+\underset{i,\sigma }{\sum }M_{i}\sigma_{z}C_{i,\sigma }^{+}C_{i,\sigma } \end{array}$$

where $C_{i,\sigma }^{+}\left(C_{i,\sigma }\right)$ is an electron creation (annihilation) operator for spin σ at site i, and t is the nearest-neighbor hopping integral. The electron hopping from j  to site i suffers an additonal phase $\emptyset_{ij}\;$ caused by the orbital effect of the magnetic field and $\emptyset_{ij}=\int_{i}^{j}A\cdot dl/\emptyset_{0}$ with the magnetic gauge potential $A=\left(0,Bx,0\right)$ and the flux quantum $\emptyset_{0}=h/e$. $M_{i}$ represents Zeeman splitting, and $\sigma_{z}$ is the Pauli matrix describing the electron spin.

The Hamiltonian of the ZBBGN under a vertical electric field and local potentials is as follows:

(2) $$H_{BBG}=H_{BMG}^{O}+H_{BMG}^{I}+t_{⊥}\underset{i\in O,j\in I}{\sum }(C_{i,\sigma }^{+}C_{j,\sigma }+H.C.)+\underset{i\in O,I,\sigma }{\sum }\varepsilon_{i}C_{i,\sigma }^{+}C_{i,\sigma }\;\begin{array}{c} +V_{1}\underset{i\in OBR,\sigma }{\sum }C_{i,\sigma }^{+}C_{i,\sigma }+V_{2}\underset{i\in OTR,\sigma }{\sum }C_{i,\sigma }^{+}C_{i,\sigma } \end{array}$$

where $H_{BMG}^{O,I}$ is the Hamiltonian of the outer (O) and inner (I) bent layers of Equation (1), and $t_{⊥}$($t_{⊥}=0.13t$ hereafter) is the interlayer hopping between the Bernal-stacked neighbors. $\varepsilon_{i}$ represents the electric potential generated by the electric field E and is expressed as $\varepsilon_{i}=-{\mathrm{eEz}}_{i}$. E is the uniform electric field along the z-axis direction, and $z_{i}$ is the z-coordinate value. For the outer bottom planar region (OBR), inner bottom planar region (IBR), inner top planar region (ITR) and outer top planar region (OTR), $z_{i}$ is taken as H/2, h/2, −h/2, and −H/2, respectively, and the electric potential $\varepsilon_{i}$ in the outer middle bent region (OMR) and inner middle bent region (IMR) varies with $z_{i}$. $V_{1}$ and $V_{2}$ are the local potentials acting on the OBR and OTR, respectively.

To study the transport properties, we calculate the two-terminal conductance using Lattice Green’s function method [33,34]. According to the Landauer–Büttiker formula, the conductance with spin σ is given by the equation $G_{\sigma }=T_{\sigma }e^{2}/h$ [19,33,35]. The transmission coefficient $T_{\sigma }$ with spin σ from lead q to lead p can be written

(3) $$\begin{array}{c} T_{\sigma }=Tr\left[\Gamma_{p\sigma }G_{\sigma }\Gamma_{q\sigma }G_{\sigma }^{+}\right] \end{array}$$

where $\Gamma_{p\sigma }\left(E\right)=i\left(\Sigma_{P\sigma }-\Sigma_{p\sigma }^{+}\right)$ is the broadening function of lead *p* with $\Sigma_{P}\;$ being the self-energy. $G_{\sigma }\left(E\right)={\left[E-H_{\sigma }^{C}-\Sigma_{P\sigma }-\Sigma_{q\sigma }\right]}^{-1}$ is the Green’s function containing sites in the central device that connect to terminals *p* and *q*, where$\;H_{\sigma }^{C}$ is the Hamiltonian of the conductor region.

As shown in Figure 1a,b, we take periodic (open) boundary conditions in the x (y) direction in numerical calculations. H (H ≈ 3.4 nm hereafter) is the interlayer distance between the OBR and OTR, and h (h ≈ 2.48 nm hereafter) is the interlayer distance between the IBR and ITR. The interlayer distances in bent bilayer graphene are 0.46 nm [36]. The system can be described by two topological equivalent geometries suffering effective electric and magnetic fields, as shown in Figure 1c–e. The electric and magnetic fields ($\vec{E}\;$ and $\;\vec{B}$) are perpendicular to the OBR, IBR, ITR, and OTR. In OMR and IMR, the effective magnetic field is the normal component of the magnetic field $\vec{B}\cdot \hat{n}$, where $\hat{n}$ is the normal direction of the nanoribbon. The corresponding magnetic flux in each hexagon is ∅=BS=0.002 in the flat region and $\emptyset =\vec{B}\cdot \hat{n}S$ in the OMR and IMR hereafter, where  S is the area of a hexagon. For the effective electric potential, we fix the electric field as E = 8.8 × 10^6^ V/m. As shown in Figure 1c, the sites of the OBR, IBR, ITR and OTR are N_1_ = 200, the sites of the OMR and IMR are N_2_ = 100 and N_3_ = 80, respectively, and the sample lengths in the y direction are denoted by Na (Na = 500) for the outer layer, corresponding to a length 53.108 nm, and Nb (Nb = 480) for the inner layer, corresponding to a length 50.978 nm, whereas the sample lengths in the x direction are infinite. To simplify, we do not consider the interlayer coupling between the sites in the IBR and ITR.

## 3. Results and Discussion

### 3.1. Edge and Interface States in the Presence of an Electric Field and Local Potential

To analyze the distribution of the edge and interface states, we diagonalize the Hamiltonian of Equation (2) and obtain the energy spectra of ZBBGR under the electric field or local potential.

**Figure 2:**
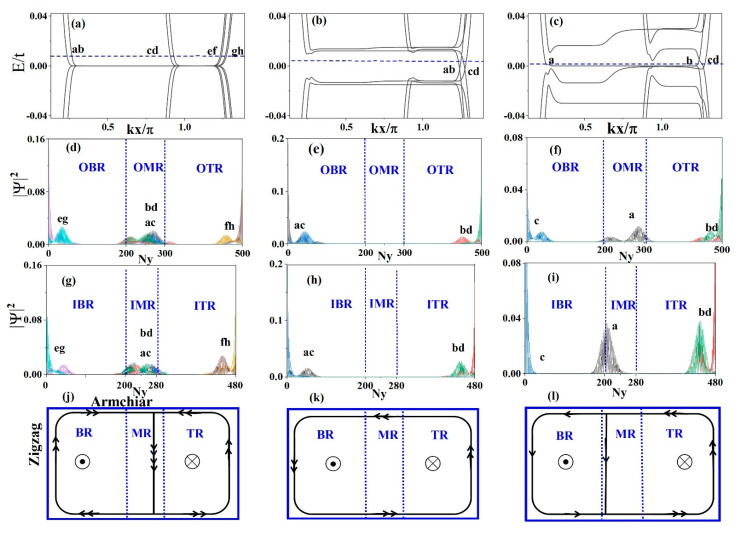
Top panels: Band structure of ZBBGN with ∅=0.002 for (**a**) E = 0, V_1_ = −V_2_ = 0; (**b**) E = 8.8 × 10^6^ V/m, V_1_ = −V_2_ = 0; and (**c**) E = 0, V_1_ = −V_2_ = 0.02t. The Fermi energy is represented by the blue dashed lines hereafter. (**d**–**f**) Second panels: the probability density distributions of edge and interface states of the outer layer indicated by letters in top panels (**a**–**c**) versus the longitudinal position index Ny. (**g**–**i**) Third panels: the probability density distributions of edge and interface states of the inner layer indicated by letters in top panels (**a**–**c**) versus the longitudinal position index Ny. (**j**–**l**) Bottom panels: schematic diagrams of edge-state propagation (the direction indicated by arrows), corresponding to the edge states marked with letters in the top panels (**a**–**c**).

When the electric field and local potential are not considered, the band structure of ZBBGN satisfies the electron-hole symmetry, as shown in Figure 2a. The probability distributions of the edge and interface states corresponding to the Fermi energy in Figure 2a are shown in Figure 2d,g. The edge states labeled by the letters e, f, g, and h are located on the boundary of the BR and TR in the inner and outer layers, respectively, while the interface states labeled by the letters a, b, c, and d are located in the MR in the inner and outer layers, respectively. These newly induced interface states in the MR are due to the change in the magnetic field direction, which eventually leads to the confinement of electrons in the magnetic interface region. The maximum degeneracy of the zeroth Landau levels (LLs) is eightfold, owing to the contribution of interface states. This is different from the electronic properties of planar bilayer graphene under a perpendicular magnetic field. These edge and interface states near zero energy are all current-carrying states.

To understand the edge-state propagation, we also need to figure out how the current flows in the y direction. Similarly, if we take periodic (open) boundary conditions in the x (y) direction, it corresponds to an armchair-edged bilayer graphene nanoribbon. The numerical results will show that the states are localized at the upper and lower edges. This means that the currents flow in the horizontal direction along both the upper and lower edges of the system.

On the other hand, because the system suffers a perpendicular magnetic field, only the edge and interface states are current-carrying states. That is to say, only the states localized at the upper and lower edges are current-carrying states in the y-direction. Therefore, when the currents flow horizontally, they will flow along both the upper and lower sides. As shown in Figure 2j, the edge currents of the BR and TR flow clockwise and counterclockwise along their boundary respectively, whereas the interface currents of the MR flow to the two plane regions.

When the electric field E is considered, the spatial inversion symmetry is broken. In addition, the electric field can lift the degeneracy of the zeroth LLs. A suitable and fixed electric field E = 8.8 × 10^6^ V/m is chosen, and the band structure and probability distribution are shown in Figure 2b,e,h. Obviously, the edge and interface states after adding the electric field are different from those in Figure 2d,g. After adding the electric field, the interface states around zero energy disappear, and the edge states appear only at the boundary of the sample. All the edge currents in the BR and TR flow counterclockwise along their boundaries, as shown in Figure 2k. This indicates that the QH phase appears in the sample, which is consistent with an unusual QH effect [37] was discovered in graphene.

When considering the local potential V_1_ = −V_2_ = 0.02t, the spatial inversion symmetry is also broken, and the band structure and probability distribution are depicted in Figure 2c,f,i. Since the local potential is only added to the OBR and OTR, some of the zeroth LLs are pushed to high energies, and the rest of the zeroth LLs are still at zero energy. Therefore, the current-carrying edge and interface states near zero energy still exist. The currents flow counterclockwise along their boundaries and interface in Figure 2l.

**Figure 3:**
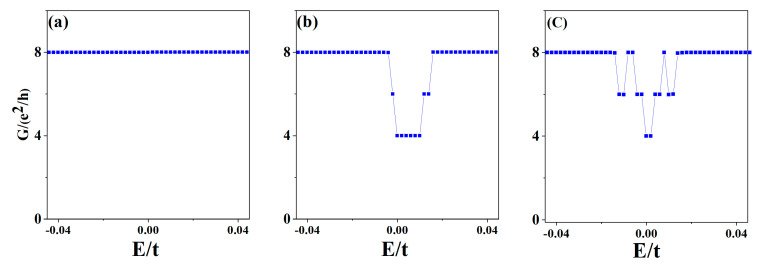
(**a**–**c**) The two-terminal conductance G as a function of Fermi energy E of Figure 2a–c.

The evolution of the LLs will lead to a change in the conductance. Using Green’s function method, we obtained the two-terminal conductance of ZBBGN under a fixed electric field or local potential. Figure 3 shows that the two-terminal conductance is quantized. When both the electric field and local potential are not considered, the quantized conductance near zero energy is G = 8e^2^/h due to the eightfold degeneracy of the zeroth LLs. Once the electric field or local potential is added, the quantized conductance around zero energy is G = 2(n + 1)e^2^/h with n = 1, 2, 3, …. The quantized conductance at zero energy is G = 4e^2^/h, because some of the zeroth LLs are pushed to high energies and the current channels are reduced by half. It is clear that both the electric field and local potential can regulate the evolution of the two-terminal conductance.

### 3.2. Zeeman Effect

**Figure 4:**
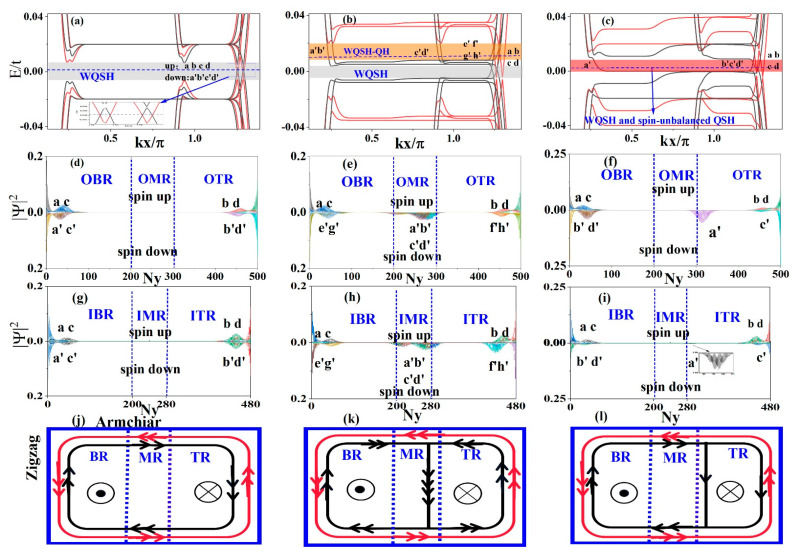
Top panels: Band structure of ZBBGN with ∅=0.002 and M = 0.02t for (**a**) E = 0, V_1_ = −V_2_ = 0; (**b**) E = 8.8 × 10^6^ V/m, V_1_ = −V_2_ = 0; (**c**) E = 0, V_1_ = −V_2_ = 0.02t. (**d**–**f**) Second panels: the probability density distributions of edge and interface states of the outer layer indicated by letters in top panels (**a**–**c**) versus the longitudinal position index Ny. (**g**–**i**) Third panels: the probability density distributions of edge and interface states of the inner layer indicated by letters in top panels (**a**–**c**) versus the longitudinal position index Ny. (**j**–**l**) Bottom panels: schematic diagrams of edge-state propagation (the direction indicate-d by arrows), corresponding to the edge states marked with letters in the top panels (**a**–**c**). The red (black) lines with arrows represent spin-up (spin-down) states.

In the presence of the Zeeman effect, the spin degenerate LLs are lifted into spin-up and spin-down branches, as depicted in Figure 4a–c, where the Zeeman splitting is M = 0.02t. We still discuss the band structures of the above three cases, but with the Zeeman effect. In Figure 4a, the sample does not suffer from the electric field and local potential, and the Zeeman field induced spin gap is Δ = 2M = 0.04t. The band structure shows a clear separation of the spin-up and spin-down energy bands compared with that without Zeeman splitting. When the Fermi energy is located in the gray region [Figure 4a], the zeroth LLs are spin-polarized as shown in Figure 4d,g. In the gray region, the helical edge states formed by counterpropagating edge states with opposite spins are located on the boundary, as shown in Figure 4j. In this case, the edge states are spin-filtered. This indicates that this is a WQSH phase characterized by two pairs of helical edge states, which is robust against nonmagnetic disorder but can be perturbed by time-reversal symmetric perturbations inducing backscattering between edge modes [20,38].

When both the fixed electric field E = 8.8 × 10^6^ V/m and Zeeman splitting M = 0.02t are considered, the spin gap is significantly reduced compared with that without Zeeman splitting, as shown in Figure 4b. When the Fermi energy lies in the gray region, the system is in a WQSH phase. Now, we focus on the spin-polarized energy band. If the Fermi energy is located in the orange region, the probability distributions of the edge and interface states are shown in Figure 4e,h. The corresponding schematic diagrams of the edge and interface state propagation are illustrated in Figure 4k, from which we find that the WQSH phase appears in the BR, whereas the QH phase appears in the TR. This indicates that the WQSH and QH phases appear in these two planar regions forming a QSH-QH junction; that is to say, the two phases can coexist in a ZBBGN system. From a topological point of view, this implies that a topological phase transition can be achieved by adjusting the electric field. In addition, the interface states in the MR are fully spin-polarized; therefore, only the spin-down current flows along the right interface. 

In Figure 4c, both the local potential V_1_ = −V_2_ = 0.02t and the Zeeman splitting M = 0.02t are taken into account, and the spin degeneracy of the band is completely eliminated. The probability distribution and flow diagrams of the edge and interface states are depicted in Figure 4f,i,l, respectively. If the Fermi energy is located in the red region EF ∈ [0, 0.01t], a WQSH phase appears in the BR whereas a spin-unbalanced QSH phase is in the TR, and the single spin-down current flows along the right boundary of the MR. Hence, the WQSH-spin-unbalanced QSH junction can be obtained in the ZBBGN. In addition, there is also a region of energy EF ∈ [−0.01t, 0] in which the WQSH phase and the spin-unbalanced QSH phase can coexist but its edge-state propagation is different from the case in Figure 4l.

**Figure 5:**
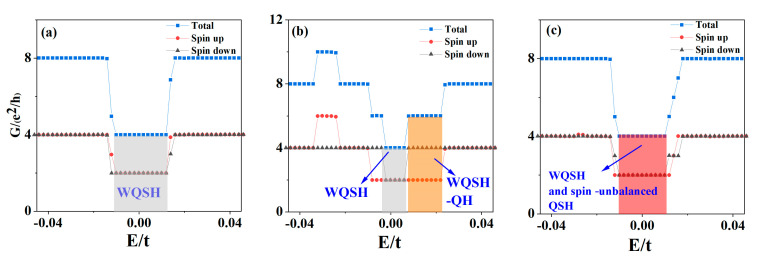
(**a**–**c**) The two-terminal conductance G as a function of Fermi energy E of Figure 4a–c.

Now let us discuss the transport properties of the above three cases. Figure 5a–c show the two-terminal conductance of ZBBGN as a function of Fermi energy. In the absence of the electric field and local potential, the total quantized conductance near zero energy is 4ne^2^/h, which is half of the quantized conductance without Zeeman splitting because the spin gap is opened and the interface states disappear. The spin-up and spin-down conductances contribute almost equally to the total conductance.

At an electric field E = 8.8 × 10^6^ V/m, the electric field leads to a band structure and quantized conductance no longer symmetric with respect to zero energy. The spin band gap is obviously smaller than that without an electric field, thus, the total conductance near zero energy is still 4ne^2^/h with n = 1, 2, 3, …. However, the total conductance is 6ne^2^/h in the orange region due to the significant spin-polarized edge and interface states. When the local potential V_1_ = −V_2_ = 0.02t is added, the band structure and quantized conductance are also no longer symmetric with respect to zero energy. Nevertheless, the local potential is only added to the BR and TR, and the spin band gap at zero energy is not fully opened. Part of the spin-up and spin-down energy bands near zero energy are shifted significantly away from zero energy, which leads to the reduction of the conductance at zero energy to 4ne^2^/h. From these results, we can see that the quantized conductance can be controlled by applying an electric field or local potential.

### 3.3. Effect of Disorder

**Figure 6:**
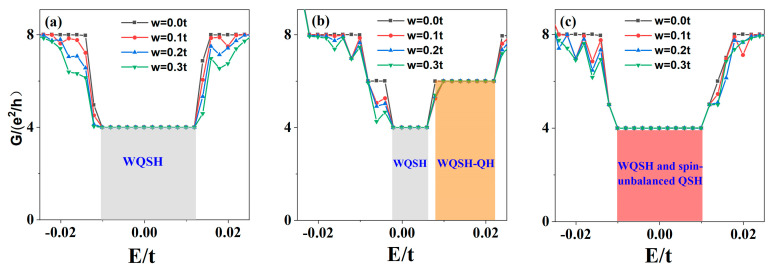
Effect of disorder on the WQSH phase. The conductance G as a function of Fermi energy E for the different disorder strengths W with ∅=0.002 and M = 0.02t for (**a**) E = 0, V_1_ = −V_2_ = 0; (**b**) E = 8.8 × 10^6^ V/m, V_1_ = −V_2_ = 0; (**c**) E = 0, V_1_ = −V_2_ = 0.02t.

Finally, we explore the stability of the WQSH phase described in the above section. First, we introduce the nonmagnetic on-site disorder potential $H_{dis}=\sum_{i}w_{i}C_{i}^{+}C_{i}$, where $w_{i}\;$ denotes the disorder potential. The distribution of the disorder potential is random, but it takes values in the range$\;w_{i}=\left[-w/2,w/2\;\right]$, where w is the disorder strength. As shown in Figure 6, we give the two-terminal conductance of the WQSH for different disorder strengths. In order to see the effect of disorder, we take the disorder strength up to ten times that of Zeeman splitting.

In Figure 6a, we find that the conductance plateaus in the WQSH phase are unbroken even up to W = 0.3t, but the higher energy plateaus are destroyed, which indicates that the WQSH effect is robust against nonmagnetic random disorder. This is similar to that of bilayer graphene [20]. Figure 6b shows that the conductance plateaus (with an electric field) in the gray and orange regions are stable even W = 0.3t >> M, which indicates that the conductance is stable in the regions where the WQSH phase or the WQSH and QH phases coexist. Moreover, the conductance of the region with both the WQSH phase and spin-unbalanced QSH phase near zero energy is also robust against nonmagnetic disorder, as shown in Figure 6c. In the above three cases, the two-terminal conductance of the WQSH phase is contributed by the edge and interface channels where the electrons are localized at the edges and interfaces, and the channels are robust against the weak nonmagnetic disorder. Therefore, the conductance of the region around zero energy is stable as long as it contains the WQSH phase.

## 4. Conclusions

In summary, we investigate the band structures and transport properties of the ZBBGN under a uniform perpendicular magnetic field using the tight-binding model and Green's function approach and propose a method to manipulate the edge and interface states by an electric field, local potential, and Zeeman splitting. When the electric fixed or local potential is added, the distribution of edge and interface states is varied, leading to the two-terminal conductance changing from 8e^2^/h to 4e^2^/h. When Zeeman splitting is considered, the edge states are spin-filtered, and a WQSH phase with two pairs of helical edge states is present. A proper electric field can make the WQSH and QH phases appear in two different planar regions of ZBBGN forming a WQSH-QH junction, while a local potential yields the coexistence of the WQSH phase and the spin-unbalanced QSH phase. In both cases, fully spin-polarized currents appear at the interface. The Zeeman effect results in the two-terminal conductance of the WQSH becoming 4e^2^/h, which is robust against weak nonmagnetic disorder. These results provide a new way to manipulate the interface states, as well as the transport properties of ZBBGN. Specifically, the ZBBGN system we studied provides a simple way to obtain fully polarized spin currents, which will have some potential applications in spintronics.

## Data Availability

The data presented in this study are contained within the article and are available on request from the corresponding author.
